# Preparing the Next Generation for STEM: Adolescent Profiles Encompassing Math and Science Motivation and Interpersonal Skills and Their Associations With Identity and Belonging

**DOI:** 10.1177/0044118X221085296

**Published:** 2022-05-03

**Authors:** Kelly Lynn Mulvey, Luke McGuire, Channing Mathews, Adam J. Hoffman, Fidelia Law, Angelina Joy, Adam Hartstone-Rose, Mark Winterbottom, Frances Balkwill, Grace Fields, Laurence Butler, Karen Burns, Marc Drews, Adam Rutland

**Affiliations:** 1 North Carolina State University, Raleigh, USA; 2 University of Exeter, Exeter, Devon, UK; 3 Cornell University, Ithaca, NY, USA; 4 Cambridge University, Cambridge, Cambridgeshire, UK; 5 Queen Mary University and Centre of the Cell, London, UK; 6 Riverbanks Zoo and Gardens, Columbia, SC, USA; 7 ThinkTank, Birmingham, UK; 8 Virginia Aquarium & Marine Science Center, Virginia Beach, VA, USA; 9 Edventure, Columbia, SD, USA

**Keywords:** science, math, social competence, adolescence, latent class analysis

## Abstract

Science, technology, engineering, and math (STEM) workers need both motivation and interpersonal skills in STEM disciplines. The aims of the study were to identify clusters of adolescents who vary in math and science motivation and interpersonal skills and to explore what factors are related to membership in a high math and science motivation and interpersonal skills cluster. Participants included 467 adolescents (312 female; *M*_age_ = 15.12 to *SD* = 1.71 year) recruited from out-of-school STEM programs in the US and UK. Findings from latent class analyses revealed four clusters, including a “High Math and Science Motivation and Interpersonal Skills” group, as well as groups that exhibited lower levels of either motivation or interpersonal skills. STEM program belonging, and STEM identity are related to membership in the high motivation and skills cluster. Findings provide insight into factors that may encourage motivation and interpersonal skills in adolescents, preparing them for STEM workforce entry.

Globally, science, technology, engineering and math (STEM) employers note a shortage of qualified, competent STEM workers with both the content knowledge and the interpersonal skills to be successful in today’s STEM workforce (National Academies of Sciences, 2016; National Science Foundation, 2015; Schneider et al., 2018). In the United States (US), STEM-related jobs are projected to grow to more than 9  million between 2014–2022 (Vilorio, 2014), and in the United Kingdom (UK), almost 50% of STEM employers report recruiting outside of the country to find skilled STEM workers (STEM Learning, 2018). Consistently, STEM employers cite a lack of core STEM skills, such as math and technology skills, but also a lack of critical skills for team-work and problem-solving among job applicants (National Research Council, 2011). There is a concerning shortage of students enrolling in STEM disciplines and subsequently becoming STEM professionals (Peterson et al., 2015), which is highlighted by the fact that growth in available STEM jobs has been three times faster than non-STEM jobs (Langdon et al., 2011). To ameliorate this shortage, not only is it imperative to foster STEM motivation, but also the interpersonal competencies that will ensure that those who enter the STEM workforce are likely to succeed. The current study aims to address this issue by identifying whether there are different clusters of adolescents in terms of both math and science motivation and interpersonal competencies. It then examines predictors of cluster membership to inform policy, programing, and practice aimed at building the STEM workforce. In terms of predictors, we attend to social factors (belonging in STEM programs), and identity-related factors (STEM identity) to comprehensively examine what is related to motivation and interpersonal competencies.

One way to address the need for STEM professionals (Peterson et al., 2015) is by ensuring that adolescents have the skills, interest, and relevant preparation to pursue STEM degrees in college and ultimately embark on STEM careers. Disparities in the representation of low-income individuals, LGBTQ individuals, women, and those from ethnic minoritized groups in STEM fields are pervasive (Freeman, 2020; National Science Board, 2018). These disparities suggest there are underrepresented groups of adolescents who could help fill the need for competent workers in the STEM workforce. Important to success in the STEM workforce is preparing adolescents with both high science and math motivation *and* interpersonal skills. It may be that social and identity-related factors will be associated with motivation and competence in adolescents who are well-prepared for entry into STEM careers. Moreover, prior research using latent class analysis documents that membership in a cluster of students with positive attitudes toward STEM did not necessarily predict STEM career attainment, especially for girls and those whose ethnic groups are typically underrepresented in STEM (Ing & Nylund-Gibson, 2013). This suggests that class analyses to understand pathways toward STEM careers should look beyond just attitudes and interest.

## Theoretical Framework

This study is guided by *social cognitive theory* (Bandura, 1992; Bandura et al., 1999), which proposes a triadic reciprocal determinism model of causality that centers on the importance of the interaction between behaviors, environment, and personal characteristics in explaining learning, and engagement. Importantly, *social cognitive theory* emphasizes the role of social environments for shaping outcomes (Bandura, 1986), recognizing that learning is an inherently social task (Vygotsky, 1978). Further our hypotheses are informed by *social identity theory* (Tajfel & Turner, 1976), highlighting the central role of feelings of belonging and identity in influencing outcomes in the lives of individuals. For example, research on occupational domains notes that motivation is especially likely to be enhanced when one holds a sense of shared social identity and receives support from one’s peers (Füllemann et al., 2015). We draw on these frameworks in considering how both interpersonal competence, and STEM motivation, can be shaped by the inherent social task of learning in distinct contexts and one’s understanding of their social identities. We focus on social factors we expect will foster interpersonal skills and motivation relevant for STEM career pursuit, specifically belonging and STEM identity.

### Adolescents Involved in STEM Programing

This study includes a sample of adolescents who are engaged in out-of-school STEM learning programs, as research suggests that out-of-school contexts may be one understudied source of both STEM motivation (Adams et al., 2015; Habig et al., 2016) and interpersonal skills (Schwarz & Stolow, 2006). Adolescents are also the focus of this study as adolescence has been shown to be a critical developmental period for fostering STEM motivation (Eccles & Wang, 2016; Wigfield et al., 2006). For example, prior research documents that participating in out-of-school programing predicts math and science motivation over time (Simpkins et al., 2006) and highlights afterschool programing as a key opportunity for social-emotional learning and the promotion of social competence (Gullotta, 2015). The participants in this study are adolescents participating in out-of-school learning programs that train youth educators. In these programs, they learn both STEM knowledge and how to interact with others, as they are trained to provide educational enrichment and to facilitate learning for visitors. Thus, we focus on a group that likely already have relatively high levels of both math and science motivation and interpersonal skills compared to their peers, although we expect to find variation even within this group of adolescents who are engaged in out-of-school STEM activities. Exploring this potential variation is a focus of the current study.

### Key Competences for STEM Success

#### Math and science motivation

Research has documented the importance of motivation for shaping one’s trajectory in science and math domains (Eccles & Wang, 2016; Wang & Degol, 2013). However, science and math motivation tend to decline across childhood and into adolescence (Frenzel et al., 2012; Jacobs et al., 2002; Muenks et al., 2018). Further, science and math motivation are essential for persistence in STEM fields of study (Graham et al., 2013). Academic motivation can be shaped by many factors, with findings suggesting that an individual’s general academic motivation is relatively stable over time, even though mean rates decline with age (Gottfried et al., 2001). However, out-of-school experiences, such as participating in afterschool programs, engaging in STEM activities with family, and visiting STEM sites such as museums, can promote math and science motivation (Liu & Schunn, 2018; Sha et al., 2016). What is still unknown, though, is whether individuals show variation in math and science motivation and, if present, what factors are associated with that variation. This is important to examine, as research notes a high degree of individual variation in motivation when measured in formal academic settings, and differences in trajectories for those who do vary in baseline motivation (Gottfried et al., 2001), but much less is known about variation in motivation amongst those who participate in out-of-school STEM programs.

#### Interpersonal skills

Whereas STEM subject motivation is clearly essential for success in addressing the global STEM workforce shortage, so too is interpersonal competence. Interpersonal competencies involve understanding, navigating, and managing social interactions with others, and have long been noted as essential for workplace success (Hayes, 2002; Klein et al., 2006). Recent reports highlight the increasing need for social skills and interpersonal competence in the workplace, with jobs requiring high levels of social interaction growing by more than 10% in recent decades (Deming, 2017) and a recent study of workers in STEM disciplines identified interpersonal skills as “highly important” for success in STEM careers (Jang, 2016). Recent research confirms key interpersonal competences for success in today’s global world, including skills relevant for teamwork, collaboration, communication, and successful interaction with others (OECD, 2018; Partnership for 21st Century Learning, 2016). Notably, the Partnership for 21st Century Learning (2016) argues that these key interpersonal skills must be integrated into all aspects of learning and that training students for success in today’s world must move beyond a sole focus on academic skills. These skills are often a key feature of out-of-school STEM programing at informal learning sites, given that these programs involve preparing youth to interact with visitors at the informal learning sites, for instance by serving as docents or interpreters.

### How to Promote Higher STEM Motivation and Interpersonal Skills

To best prepare individuals for entry into the STEM workforce, programs aimed at enhancing adolescents’ interest in STEM need to build STEM motivation and interpersonal competence concurrently. To date, little is known about whether adolescents vary in terms of their interpersonal skills and STEM motivation. However, understanding whether there are different clusters of adolescents in regard to interpersonal skills and STEM motivation, as well as what is associated with membership in these clusters, may provide important insight into understanding what is important in the dynamic between interpersonal skills and STEM motivation. Recent research suggests that out-of-school STEM programs may play a role in shaping optimal outcomes as feelings of belonging within out-of-school STEM communities are associated with science and math self-efficacy and interest (Hoffman et al., 2021). Thus, an aim of the current study is to examine associations between membership in a cluster with high science and math motivation and high interpersonal competence, with attention to belonging and identity, as these have been noted as key factors that promote academic motivation and social competence (Muenks et al., 2018).

#### Belonging

Research on school belonging highlights the importance of feeling like one “fits-in” for success in school (Cemalcilar, 2010; Juvonen, 2006; Neel & Fuligni, 2013). Further, a sense of belonging has been documented as critical for youth who may be typically marginalized from STEM domains (Chang et al., 2014; Cheryan et al., 2017; Graham et al., 2013; Rattan et al., 2018). Most of the research on belonging has centered on formal school contexts (Juvonen, 2006) however youth often have the opportunity to be part of additional learning communities outside of school (Peppler, 2017) and research highlights the importance of out-of-school spaces for building belonging (Akiva et al., 2013; Hoffman et al., 2021). Findings suggest that out-of-school programs can foster belonging through strong connections to the program leaders and that belonging is enhanced for youth who spend more time participating in these out-of-school programs (Akiva et al., 2013). Research suggests that high quality out-of-school programs can support youth development (Kahne et al., 2001) and that interpersonal skills and relationships are central to feelings of belonging (Baumeister & Leary, 1995); however less is known about how the sense of belonging within these out-of-school learning contexts is related to STEM motivation and interpersonal skills for youth.

#### STEM identity

In addition to belonging, we expect that STEM identity may be related to adolescents’ developing motivation and interpersonal skills. Scholars have posited that identifying with STEM (i.e. thinking of STEM as a central part of one’s self-concept) may be a key predictor of long-term persistence in STEM domains (Graham et al., 2013). STEM identity may develop through formal encounters with STEM, and out-of-school learning experiences (Goff et al., 2020; National Research Council, 2009). Further, STEM identity may be an important predictor of motivation more generally (Robinson et al., 2019; Schinske et al., 2016; Starr, 2018). Positive social interactions around STEM foster STEM identity (Kim et al., 2018), suggesting there may be a relationship between STEM identity and interpersonal competence. Research, however, has not fully explored the associations of STEM identity with both STEM motivation and interpersonal skills among adolescents.

## Current Study

Given the importance of both math and science motivation and interpersonal competence, the aim of the current study is to explore whether there were different clusters or classes of adolescents based on math and science motivation, and interpersonal skills. Additionally, we aim to identify whether belonging in a STEM program, and STEM identity, were related to membership in a high interpersonal skills and math and science motivation cluster, as compared to other clusters. We use a person-centered approach (latent class analysis) for this study as person-centered approaches can provide key insight not afforded by variable-centered approaches to analyses (Bergman & Trost, 2006). In particular, latent class analysis allows us to identify subgroups within a sample that help to characterize the ways in which a sample is heterogenous (Nylund-Gibson & Choi, 2018). Given that adolescents who participate in out-of-school STEM programs often demonstrate strong STEM motivation (Adams et al., 2015; Habig et al., 2016; Simpkins et al., 2006), as well as social competence (Hoffman et al., 2021), we expect to identify a cluster of adolescents with high math and science motivation as well as high interpersonal skills. However, given that not all youth who engage in afterschool programing report positive outcomes (Ing & Nylund-Gibson, 2013) and that findings suggest high inter-individual variation in constructs such as motivation (Gottfried et al., 2001), we also expect to document other clusters of students who vary on these two dimensions, including a cluster that is lower on both motivation and interpersonal skills. Membership in the high cluster would position adolescents well for successful entry into the STEM workforce. Thus, we also propose that those who hold membership in this cluster as compared to other clusters may differ in belonging and STEM identity. Specifically, given prior research documenting the role of belonging and identity for STEM outcomes (Eccles & Wang, 2016; Hoffman et al., 2021; Leaper et al., 2012) and social competence (Baumeister & Leary, 1995), we expect that adolescents who reported greater STEM program belonging, and a stronger STEM identity would be more likely to be in the high motivation and interpersonal competence cluster.

## Method

### Participants

Our sample included 467 adolescents (312 female, 153 male, and nine who did not identify their gender; *M*_age_ = 15.12 to *SD* = 1.71 year), recruited from the United States (*n* = 291) and the United Kingdom (*n* = 176). Participants reflected the ethnic-racial diversity of their communities, with 44.4% White British or European American, 20.7% Asian British or Asian American, 15.6% Black British or African American, and 16.2% bi-racial or other. 3.1% of participants chose not to report their ethnicity. Participants were recruited through informal STEM learning sites, where all participants were starting youth educator programs that involved learning about STEM content and serving as educators or docents who would share STEM content and experiences with visitors (for instance, by staffing exhibits). Participants in the US were youth educators at either a zoo (13%), a children’s museum (9.3%) or an aquarium (39.8%). Participants in the UK were youth educators at either a science and technology museum (10%), a medical history museum (3.7%), or a cell biology science education center (24.1%).

### Procedure

Participants were part of a larger study of adolescents’ STEM orientation and STEM-related outcomes. The study was approved by the IRB at the **University of Exeter** in the UK and **North Carolina State University** in the US. All parents received notification of the study and all participants assented to participation. Participants were compensated with a small gift card. Participants completed the survey independently on a computer within the first month of their time in the youth program.

### Measures

#### Math and science motivation

Math and science motivation was measured using a measure adapted from Wang, Eccles et al. (2013). For each domain (math and science), participants completed 11 Likert-type items capturing math and science motivation (1 = *Not at all true*; 7 = *Very true*; α = .89). Items include measures of self-concept, interest, and expectancies, for instance: “How good would you be at learning something new in math?” and “How much do you like science?” Composite scores were created across domains.

#### Interpersonal competence

Interpersonal competence was assessed with a measure of social and interpersonal competence (Marsh et al., 1983). The measure assesses the extent to which youth believe they have the skills and competencies to engage with their peers. This six item Likert-type measure has been used to measure interpersonal competence in individuals from early childhood through emerging adulthood. An example item reads, “I make friends easily” (1= *Not at all true*; 4 = *Very true*). Composite scores were created, where higher scores indicated higher levels of interpersonal skill (α = .86).

#### Belonging in STEM program

To measure youths’ belonging in their STEM program, we used an adapted version of the Mendoza-Denton et al. (2002) Institutional Belonging scale, which measured students’ belonging within their STEM major. In this adapted version, items were edited to focus on belonging to one’s STEM youth educator program. The scale consisted of eight Likert-like items. An example item from the scale reads, “How much do you feel that you fit in within your specific STEM program?” (1 = *Definitely do not fit in*; 10 = *Definitely fit in*). Composite scores of belonging to the STEM program were created with higher scores indicated higher levels of belonging (α = .95).

#### STEM identity

As a measure of participants’ STEM identity, adolescents were asked to select one image from a series of seven increasingly overlapping pair of circles that best represents their STEM identity. One circle in each pair read “you” and the other circle read “STEM.” To capture how much one perceives STEM to be integrated into one’s sense of self, adolescents were asked, “Please look carefully at these pictures and then answer the question below. Select one of the seven pairs of overlapping circles shown below that best represents how compatible you think your two identities are.” Higher scores represent a stronger STEM identity and lower scores represent a weaker integration of STEM identity into the sense of self (1 = no integration to 7 = full integration). This pictorial measure was adapted from a well-established measure of integration of identities, the “Inclusion of Other in the Self” measure, which has been used across a range of age groups and domains (e.g., London et al., 2011; Tropp & Wright, 2001).

### Data Analytic Plan

First, LCA using Latent GOLD 5.1 (Vermunt & Magidson, 2016) was estimated to identify underlying classes (or clusters) of participants based on math and science motivation and interpersonal competence. Once clusters were identified and individuals assigned using modal assignment, a multinomial logistic regression was conducted to examine whether STEM Program Belonging, and STEM Identity predict membership in the different clusters. Listwise deletion was used to account for missing data. Please see Table 1 for correlations between variables and Table 2 for descriptive statistics.

**Table 1.** Correlations.

	1.	2.	3.	4.	5.	6.	7.	8.
1. Age								
2. Gender (1 = Male and 2 = Female)	.09*							
3. Country (0 = USA and 1 = UK)	.46**	.23**						
4. Ethnic Majority Status (1 = Majority and 2 = Minority)	.17**	.07	.49**					
5. Math and Science Motivation	−.03	−.05	.07	0.01				
6. Interpersonal Skills	−.11	−.01	−.30**	−.17**	.13*			
7. Belonging	−.10	−.10	−.07	−.03	.23**	.30**		
8. STEM Identity	−.07	−.10	−.20**	−.13	.40**	.13	.21**	

* *p* <0.05, **, *p* < .01.

**Table 2.** Descriptive Statistics.

Variable	Mean (*SD*)	Skewness (*SE*)	Range
Math and science motivation	61.40 (7.42)	−0.82 (0.12)	11–77
Interpersonal skills	3.06 (0.64)	0.79 (0.15)	1–4
Belonging	8.12 (1.59)	−1.03 (0.18)	1–10
STEM identity	5.34 (1.86)	−0.73 (0.17)	1–7

## Results

### Clusters Based on Math and Science Motivation and Interpersonal Skills

We compared latent class models with different numbers of clusters (1–8) and assessed the comparative model fit to identify the most parsimonious model while also explaining the greatest amount of association among the variables. We assessed model fit by examining the Bayesian Information Criterion (BIC), and the likelihood ratio chi-squared statistic (L^2^), as well as a non-significant *p-*value (*p* > .05). Models with lower BIC and L^2^ values indicate a better fit. Finally, the bivariate residuals were examined to ensure that all bivariate residuals are lower than 3.84 (Vermunt & Magidson, 2016).

All models tested converged. Model fit statistics are presented in Table 3. Both the three and four-class model had good fit. Thus, we used conditional bootstrapping to compare the model fit between the three- and four-class models by assessing the difference in *L*^2^ values for both models. The four-class model provided a significant improvement in fit over the three-class model (*p* = .04). Further, the four-class model had comparatively low BIC and *L*^2^ values, a non-significant *p*-value, very low bivariate residuals (below .15) and clusters that were theoretically justified. Thus, we selected the four-class model.

**Table 3.** Model Fit Statistics for Latent Class Analyses.

	BIC	*L* ^2^	*Df*	*p*-Value
One class	2,956.369	110.9294	80	.013
Two class	2,965.634	101.7109	77	.031
Three class	2,967.981	85.5742	74	.17
Four class	2,978.215	77.3246	71	.28
Five class	2,993.045	73.6702	68	.3
Six class	3,014.657	76.7995	65	.15
Seven class	3,028.407	72.0652	62	.18
Eight class	3,046.579	71.7535	59	.12

The first cluster in the four-class model (56.8%; *n* = 269) is the “Lower Motivation and Interpersonal Skills” Cluster. Participants in this cluster were lower on math and science motivation as well as on interpersonal competence as compared to the other clusters, although they were still above the midpoint on both constructs, see Table 4. The second cluster (35.4%; *n* = 168) reflects “High Motivation and Interpersonal Skills” as this group reported high motivation and relatively high interpersonal skills. The third cluster (3.6%; *n* = 17) reflects participants who were “High Motivation and Low Interpersonal Skills,” reporting high math and science motivation, but the lowest interpersonal competence amongst all the groups. The fourth cluster (4.2%; *n* = 20) “Lower Motivation and High Interpersonal Skills” reported the lowest rates of math and science motivation and the highest interpersonal competence.

**Table 4.** Mean Scores (and Standard Deviations) for Each Cluster.

	Lower motivation and interpersonal skills *N* = 269	High motivation, and interpersonal skills *N* = 168	High Motivation and low interpersonal skills *N* = 17	Lower motivation and high interpersonal skills *N* = 20
Motivation	56.40 (6.10)^a^	67.42 (3.26)^a,b^	67.61 (2.75)^a,c^	55.10 (4.68)^b,c^
Interpersonal skills	2.70 (0.34)^d^	3.38 (0.54)^d,e^	2.28 (0.22)^d,e,f^	4.10 (0.20)^d,e,f^

*Note*. Science and Math motivation were measured on a scale from 0 to 77; Interpersonal competence was measured from 1 to 4, pairs marked with the same superscript letter differ significantly at *p* < .001.

Importantly, across the board, participants were above the mid-point for both math and science motivation as well as for interpersonal competence although there was meaningful variation. ANOVA analyses documented that there were significant differences across clusters in motivation (*F*[3, 396] = 168.004, *p* < .001, *η_p_*^2^ = 0.56) and interpersonal skills (*F*[3, 256] = 104.01, *p* < .001, *η_p_*^2^ = 0.55), see Table 4 for pairwise comparisons.

Next, chi-square analyses were conducted to assess whether there were differences in cluster membership based on gender, majority ethnic status, and country. Findings revealed that there were no differences based on gender (*χ*^2^ [3] = 1.97, *p* = .57), or majority ethnic status (*χ*^2^ [3] = 2.60, *p* = .47). However, more participants from the US and fewer participants from the UK than expected were in the High Motivation and Low Interpersonal Skills cluster, (*χ*^2^ [3] = 12.82, *p* = .005). There were no differences by country on the other three clusters.

#### Predictors of cluster membership

Next, in order to test which factors predict high motivation and high interpersonal competence, we performed a multinomial logistic regression analysis using cluster membership as a categorical dependent variable. Gender, ethnicity (majority/minority), country, belonging, and STEM identity were predictors of cluster membership (with the High Math and Science Motivation, and Interpersonal Competence cluster as the reference group). Results indicated that the more participants reported belonging with their STEM program (OR: 0.68, χ^2^[1] = 9.24, *p* = .002) and the greater their STEM Identity (OR: 0.62, χ^2^[1] = 16.98, *p* < .001) the less likely participants were to be assigned to the Lower Motivation and Interpersonal Skills cluster, as compared to the Higher Motivation and Interpersonal Skills cluster. Further, the more participants reported belonging with their STEM program (OR: 0.67, χ^2^[1] = 4.068, *p* = .044) the less likely participants were to be assigned to the High Motivation and Low Interpersonal Skills cluster, as compared to the Higher Motivation and Interpersonal Skills cluster. Finally, the greater their STEM identity (OR: 0.58, χ^2^[1] = 4.028, *p* = .045) the less likely participants were to be assigned to the Low Motivation and High Interpersonal Skills cluster, as compared to the Higher Motivation and Interpersonal Skills cluster. Thus, belonging and STEM identity are important predictors of which adolescents will be in the high motivation and interpersonal skills cluster.

## Discussion

This study reveals heterogeneity in math and science motivation, and interpersonal competence among adolescents who are involved in out-of-school STEM youth educator programs. This is especially important to document as youth involved in these programs might be expected to all have high levels of both STEM motivation and interpersonal skills, given that the programs require STEM educational outreach to the visitors at the STEM learning sites. Success with this type of educational outreach would be fostered by both high math and science motivation and interpersonal competence. What we document, however, is that only a minority of adolescents sampled (35.4%) were part of the High Math and Science Motivation and Interpersonal Competence cluster. Moreover, the results highlight important ways in which STEM program belonging, and STEM identity are related to membership in this cluster. These findings suggest that practitioners should focus on building both math and science motivation and interpersonal competence, as most adolescents in this study do not fall into the cluster that reflects high levels of both. Further, practitioners might focus on honing belonging and STEM identity as ways to help enhance motivation and competence.

In our first aim, we document a cluster of adolescents who possess both high math and science motivation and interpersonal competence; however, this cluster did not represent the majority of adolescents. More than half of adolescents were represented in a cluster with lower motivation and interpersonal competence. Though these adolescents did have lower levels of motivation and competence, they were still relatively high on both variables, which may be because they took part in programs at informal STEM learning sites that explicitly cater to adolescents who express STEM motivation and who want to interact with visitors to these sites (and thus, might be expected to have strong interpersonal competence). There is, therefore, variation in adolescents’ motivation and interpersonal skills even in a group that might (in theory) be high on both, given that they have selected to participate in youth educator programs in informal STEM learning sites.

In our second aim, we sought to uncover what is associated with membership in the high motivation and interpersonal competence cluster, as compared to the lower motivation and interpersonal competence cluster. We focused on both social and identity-related factors to capture a more comprehensive picture of what factors could be targeted for intervention. We found that belonging was a key predictor: participants who reported greater belonging with their STEM program were less likely to be assigned to the lower motivation and interpersonal skills group or the high motivation and low interpersonal competence cluster than to the high interpersonal skills and motivation group. This extends prior research documenting the importance of formal school belonging (Cemalcilar, 2010; Faircloth & Hamm, 2005; Juvonen, 2006), as well as work showing that belonging is related to math and science interest and efficacy (Hoffman et al., 2021), finding that out-of-school program belonging is associated with math and science motivation and interpersonal competence. Prior research has noted that interpersonal connections with program leaders are critically important for STEM out-of-school programing (National Research Council, 2015) and findings suggest that afterschool or out-of-school programs can foster belonging through strong connections to the program leaders (Akiva et al., 2013). Given the present findings, which document the role of belonging in STEM program for youth who exhibit high motivation and interpersonal skills, programs should aim to build belonging and create a strong community amongst participants.

We also document that stronger STEM identity was associated with membership in the high cluster as compared to the lower motivation and interpersonal competence cluster or to the low motivation and high interpersonal competence cluster. This affirms the relation of STEM identity with the skills necessary to persist and enter the STEM workforce, which has been documented more often with college students than adolescents (Flowers & Banda, 2016; Merolla & Serpe, 2013; Starr, 2018). It also suggests that programs might work to promote a sense that STEM is “for you” in order to encourage adolescents to build their sense of competence in STEM domains. Promoting STEM identity may be an especially important avenue for intervening with adolescents from groups who are traditionally underrepresented in the STEM fields, as prior research in the US and the UK has shown that women, ethnic minority youth, and LGBTQ + youth may have more difficulty in seeing STEM as part of their identity (Archer et al., 2015; Cheryan et al., 2017; National Girls Collaborative Project, 2016; Starr, 2018).

Our findings extend prior research on youth in STEM out-of-school learning programs by using person-centered analyses, which provide additional insight beyond traditional variable-oriented approaches (Bergman & Trost, 2006), and document that fostering both feelings of belonging to STEM program and STEM identity may be important ways to encourage the development of motivation and competence. Research has suggested that out-of-school contexts can be especially beneficial for one’s sense of belonging (National Research Council, 2009) and prior evaluations of out-of-school STEM learning programs for teens have noted the benefits of these programs in promoting STEM motivation (Association of Science Technology Centers, 2000; Sneider & Burke, 2010), STEM interest and understanding (Liu & Schunn, 2018; National Research Council, 2015) and STEM career trajectories (Habig et al., 2016; National Research Council, 2015), noting as well the importance of building interpersonal relationships with program leaders (Price et al., 2019). Our findings extend this prior work by documenting that youth who are involved in out-of-school STEM programs are not homogenous in their STEM motivation and interpersonal skills. This suggests that adolescents, even those involved in out-of-school programing that promotes STEM knowledge, may not have the STEM motivation and interpersonal skills needed to prepare them for the STEM workforce. We argue that encouraging participation in, and sense of belonging with, out-of-school STEM programing, with particular attention to building STEM identity, can help to foster key skills and motivation in both areas.

## Limitations

Although this work provides important insight in documenting distinct clusters of youth across two countries who vary in their STEM motivation and their interpersonal competence, there are some limitations of the study. First, we controlled for country, gender, and ethnicity and did not document any differences in associations with cluster membership based on these characteristics. More participants from the US and fewer participants from the UK than expected were in the High Motivation and Low Interpersonal Skills cluster. Though this is intriguing, there were only a small number of participants represented in this group, generally, so interpreting this finding is not straightforward. We did not find differences in cluster membership based on gender or majority ethnic background, but our sample was too small to carefully explore differences for adolescents from different ethnic backgrounds. We were only able to examine ethnic minority versus ethnic majority youth, but there may be interesting differences within different ethnic minority groups in cluster membership as well; for instance, Black American adolescents may differ from Black British adolescents or from Asian American adolescents given the different cultural and national heritage factors they experience within the US and UK contexts. Additionally, we focused on adolescents who might have or be developing higher math and science motivation and interpersonal skills because they are participating in programs that rely on these skills. It would also be interesting to examine a broader population of youth who are, and are not, involved in out-of-school STEM programing to better understand the generalizability of the clusters found in the current study. Finally, participants were surveyed early in their program experience. It will be important for future research to follow up with youth or to examine patterns of change in cluster membership longitudinally. Longitudinal research can also test causal patterns in predicting cluster membership, which we were unable to do with our data. For example, future research might use latent transition analyses to understand whether adolescents who participate in out-of-school STEM programing maintain their cluster membership or if more individuals move into a high motivation and interpersonal skills cluster over time. Such associations are critical to evaluate the effectiveness of STEM based programing in fostering motivation and interpersonal skills that are critical in youth development.

## Conclusion

The current study documents important variation in adolescents’ math and science motivation and interpersonal competence and highlights key social factors that might be important targets for intervention to foster future interest and success in the STEM workforce. Namely, our findings suggest that interventions and out-of-school STEM programs might focus on building belonging with STEM programs, and STEM identity to enhance math and science motivation and interpersonal skills. Globally, STEM employers are seeking applicants who have high levels of both STEM and social competence (National Research Council, 2011), yet formal school environments often place greater focus on building core knowledge in STEM domains than on fostering soft skills such as interpersonal competence, communication, and collaboration skills, despite research documenting how important social emotional learning is for success (Taylor et al., 2017). Thus, to best prepare our adolescents for entry into the STEM workforce, we argue that educators should foster both high STEM motivation and interpersonal competence. This will ensure that adolescents are equipped with the interpersonal competence and the motivation that are needed to be successful in a global STEM workforce.
